# STK405759 as a combination therapy with bortezomib or dexamethasone, in *in vitro* and *in vivo* multiple myeloma models

**DOI:** 10.18632/oncotarget.25825

**Published:** 2018-07-31

**Authors:** Gabriela Rozic, Lena Paukov, Ziv Cohen, Irit Shapira, Adrian Duek, Ohad Bejamini, Abraham Avigdor, Arnon Nagler, Igor Koman, Merav Leiba

**Affiliations:** ^1^ Ariel University, Ariel, Israel; ^2^ Division of Hematology and BMT, Sheba Medical Center, Tel Hashomer, Ramat Gan, Israel; ^3^ Tel Aviv University, Sackler School of Medicine, Tel Aviv, Israel; ^4^ Division of Hematology, Assuta, Ashdod University Hospital, Faculty of Health Sciences, Ben Gurion University of the Negev, Beersheba, Israel

**Keywords:** multiple myeloma, STK405759, dexamethasone, bortezomib, survival

## Abstract

Multiple myeloma (MM) remains an incurable hematological malignancy. Combination regimens of conventional and novel drugs have improved patient’s survival. However, most patients inevitably relapse and become refractory to the current therapeutic armamentarium.

We investigated the efficacy of combining the microtubule-targeting agent STK405759 with dexamethasone or bortezomib *in vitro* and *in vivo*.

STK405759 combined with dexamethasone or bortezomib had synergistic cytotoxic activity in RPMIS, CAG and MM1.S human MM cell lines through activation of caspase 2, 3, 8, 9 and PARP. These treatments remained cytotoxic in the presence of bone marrow stroma cells. In other MM cells, including cells resistant to vincristine, melphalan, mitoxantrone or dexamethasone, these combinations decreased significantly survival as compared to single agents.

In *in vivo* studies, STK405759 disrupted existing blood vessels in xenograft tumors, acting not only as a cytotoxic agent but also as an anti-angiogenic drug. Mice treated with STK405759 in combination with dexamethasone or bortezomib resulted in greater tumor growth inhibition, increased overall response and prolonged survival as compared to as compared to BTZ or DEXA alone. Their anticancer activity was mediated by activation of apoptosis and reduction of tumor microvessel density.

These preclinical studies provide the rationale for future clinical trials of STK405759, dexamethasone and bortezomib combinations to improve the outcome of multiple myeloma patients.

## INTRODUCTION

Multiple myeloma (MM) is a tumor of clonal plasma cells in the bone marrow, often associated with bone lesions, kidney dysfunction, impaired immunity, and anemia. Multiple myeloma accounts for 1.5% of all cancers and approximately 13% of all hematologic malignancies [1].

The introduction of novel drugs, including immunomodulatory (lenalidomide, pomalidomide), proteasome inhibitors (bortezomib [BTZ], carfilzomib, ixazomib), and more recently, the deacetylase inhibitor panobinostat and the monoclonal antibodies daratumumab and elotuzumab, have improved outcomes [2–4]. However, most patients eventually relapse and become resistant to current therapies. The prognosis of MM patients who have received at least three prior lines of therapy is very poor, with event-free survival and overall survival of only 5 and 13 months, respectively [5, 6]. Hence, the pursuit for newer classes of drugs and novel therapeutic approaches is critical, especially in the high-risk and relapsed-refractory settings.

The use of microtubule-targeting agents (MTAs), has significantly increased response and survival among patients with many cancers including solid tumors and hematological malignancies [7–9]. The tubulin depolarizing agent vincristine has been used largely in different combination regimens for refractory MM [10]. It is effective until patients develop drug resistance or side-effects related to neurological toxicity [11–14]. Therefore, developing MTAs that are less toxic, more potent and can overcome drug resistance, represents a promising strategy for MM therapy.

Recently, we showed that STK405759 is a tubulin depolymerizing agent with potent, selective cytotoxic activity against myeloma cells. STK405759 as a single agent, reduced tumor burden and increased survival of treated mice in a MM xenograft model [15].

In the present study, we investigated the efficacy and toxicity of STK405759 in combination with dexamethasone (DEXA) or with the proteasome inhibitor BTZ in resistant MM cell lines and in a xenograft mice model.

## RESULTS

### STK405759 in combination with DEXA or BTZ reduces viability of MM cells

Previously, we showed *in vitro* synergistic cytotoxic activity between STK405759 and DEXA or BTZ in human RPMI-S and MM.1S cell lines [15]. Here, we extended their effect to a larger spectrum of MM cells.

**Figure 1:**
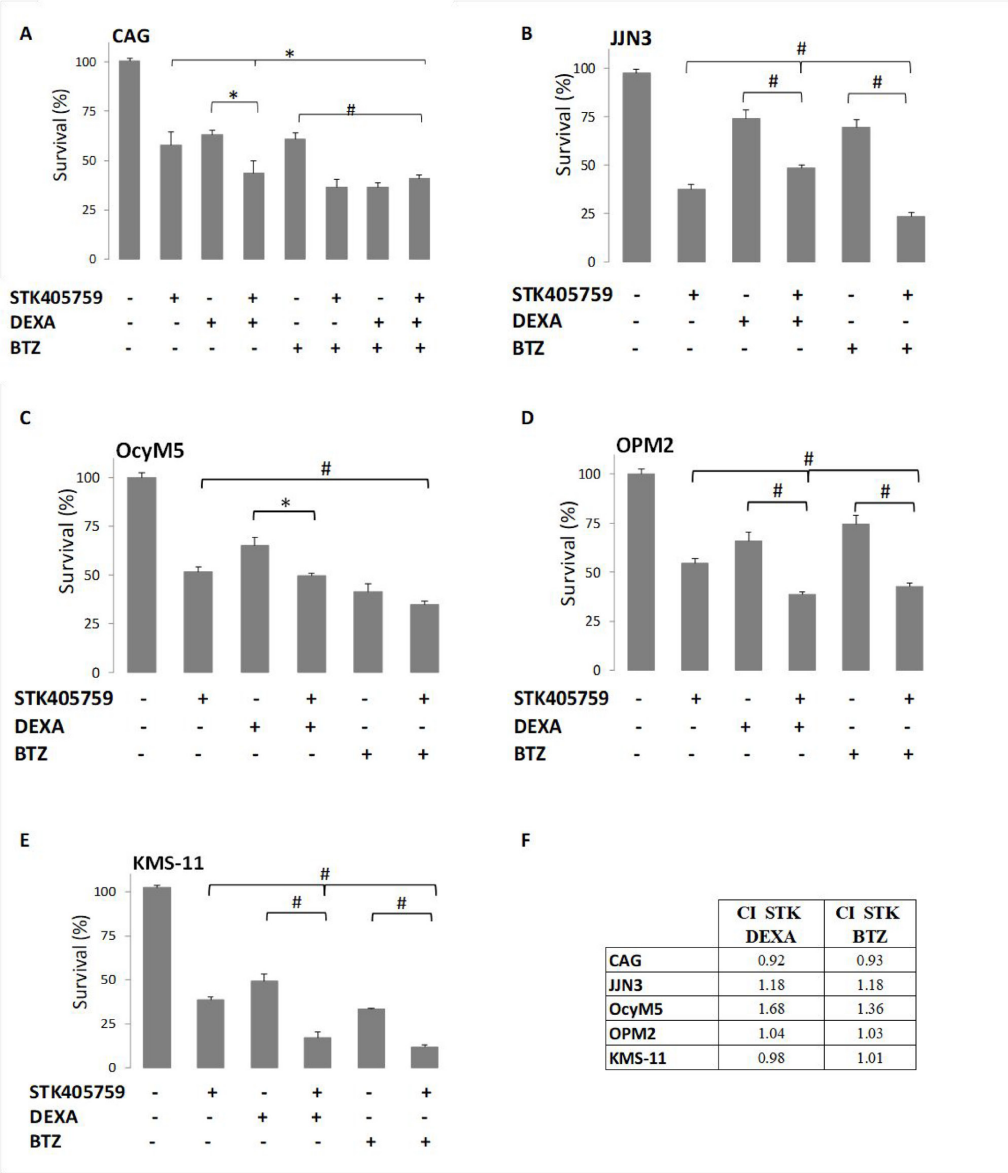
STK405759 combined with DEXA or BTZ demonstrated enhanced cytotoxicity in MM cells as compared to single agents Viability of cultured cells treated with STK405759, DEXA, BTZ or combined STK405759 with DEXA or BTZ for 48 h was assessed by XTT assay in (**A**) CAG, (**B**) JJN3, (**C**) OcyM5, (**D**) OPM2, and (**E**) KMS-11MM cell lines (**F**) STK405759 combinatorial effect with DEXA or BTZ. Combination index (CI) was calculated using compusyn software. Each treatment was performed in triplicate in three independent experiments and presented as mean ± SE. Values were normalized to the drug-free control. Results are presented as mean ± SE. ^*^p < 0.05; ^#^*p* < 0.0001 *t*-test.

**Figure 2:**
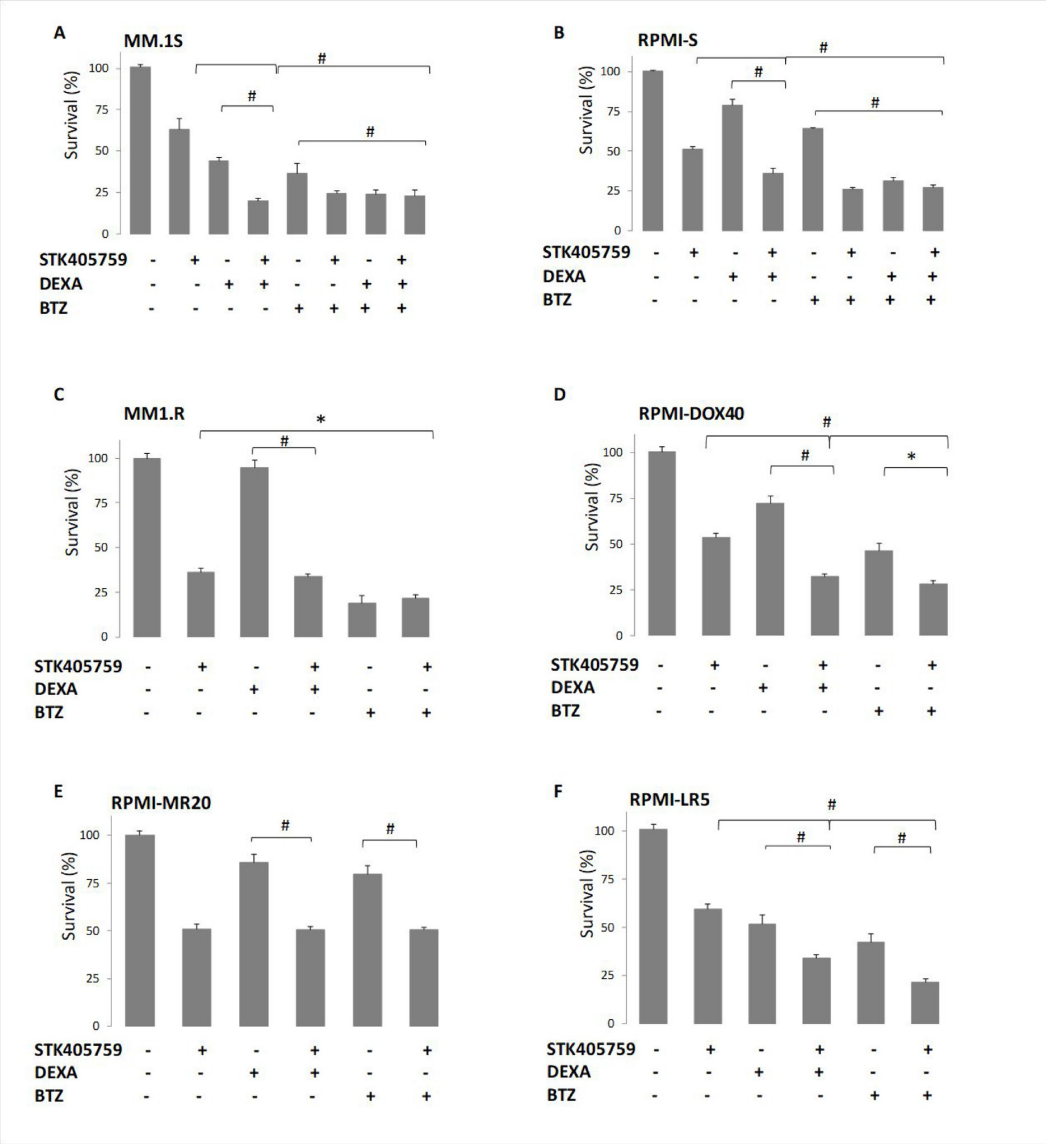
MM resistant cell lines responded to STK405759 with DEXA or BTZ cytotoxic activity Viability of cultured cells treated with STK405759, DEXA, BTZ or combined STK405759 with DEXA or BTZ for 48 h was assessed by XTT assay in (**A**) MM.1S, (**B**) MM.1R, (**C**) RPMI-S, (**D**) RPMI-DOX40, (**E**) RPMI-LR5 and (**F**) RPMI-MR20 resistant cell lines. Each treatment was performed in triplicate in three independent experiments and presented as mean ± SE. Values were normalized to the drug-free control. Results are presented as mean ± SE. ^*^p < 0.05; ^#^*p* < 0.0001 *t*-test.

STK405759 exposure in combination with DEXA or BTZ significantly reduced survival of CAG, JJN3, OcyM5, OPM2 and KMS-11 MM cells, as compared to single drugs, even when cells were minimally responsive to the single agent. However, this effect was synergistic only in CAG cells (CI: 0.9). Importantly, low concentrations of STK405759 combined with DEXA or BTZ treatments of the melphalan (RPMI-LR5) and vincristine resistant (RPMI-DOX40) cell lines showed enhanced cell death as compared to each drug alone. In contrast, when treated with combined DEXA or BTZ and STK405759, cells resistant to DEXA (MM1.R) and mitoxantrone, (RPMI-MR20) showed a cytotoxic response similar to STK405759 treatment alone (Figures 1–2). The triple combination of STK405759 with DEXA and BTZ didn’t changed the cytotoxicity as compared to STK405759-DEXA, STK405759-BTZ or DEXA-BTZ treatments in RPMI-S, MM1.S and CAG cells.

### STK405759 in combination with DEXA or BTZ overcomes bone marrow resistance

**Figure 3:**
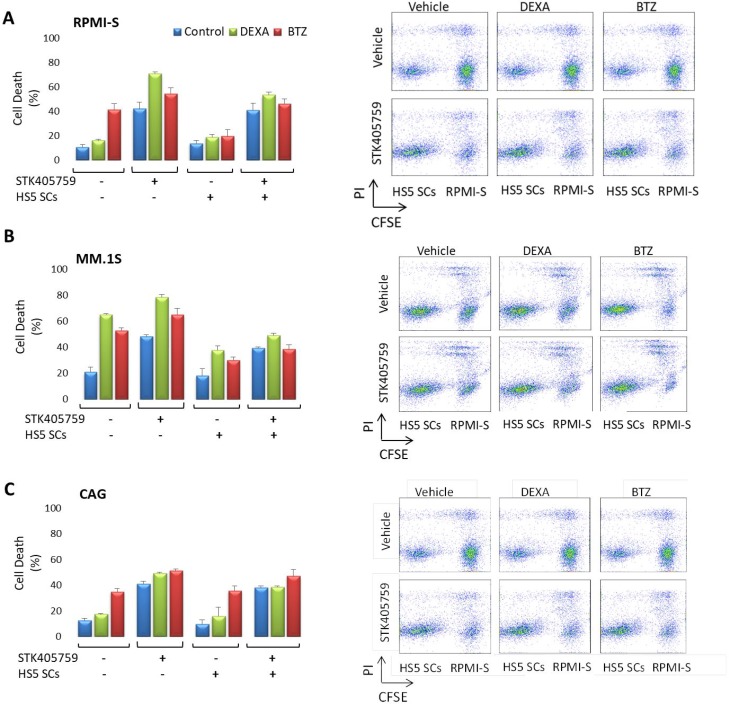
MM cells were sensitive to STK405759 in combination with DEXA or BTZ in the presence of bone marrow stoma cells (**A**) RPMI-S, (**B**) MM1.S and (**C**) CAG cells, were stained with CFSE, co-cultured with HS-5 and exposed to STK405759, DEXA and BTZ alone or in combination with STK405759 for 48 h. The cells were counterstained with PI to distinguish live from nonviable cells using FACS analysis. (B) The values of the fraction of nonviable MM cells cultured alone or with HS-5 stromal cells are presented as a function of treatment. Data presented are from three independent experiments and presented as mean ± SE. ^*^p < 0.05; ^#^*p* < 0.0001 *t*-test.

The MM-host bone marrow microenvironment confers growth, survival and drug resistance to MM cells [16]. RPMI-S, MM.1S and CAG MM cells were stained with CFSE, co-cultured with HS5 bone marrow stroma cells, and treated for 48 h with the different drugs and their combinations. Cell viability was then analyzed with propidium iodide. HS5 cells decreased the cytotoxicity of DEXA and BTZ on RPMI-S and MM.1S cells, but not of STK405759. CAG co-cultured with HS5 cells were sensitive to BTZ and STK405759, but not to DEXA. The combination of STK405759 with DEXA or BTZ increased their cytotoxicity on MM co-cultured cells, as compared with STK405759 alone. HS5 viability was not affected by any treatment in co-cultured cells (Figure 3).

### Cytotoxic mechanisms mediating the anti-MM activity of STK405759 plus DEXA or BTZ

**Figure 4:**
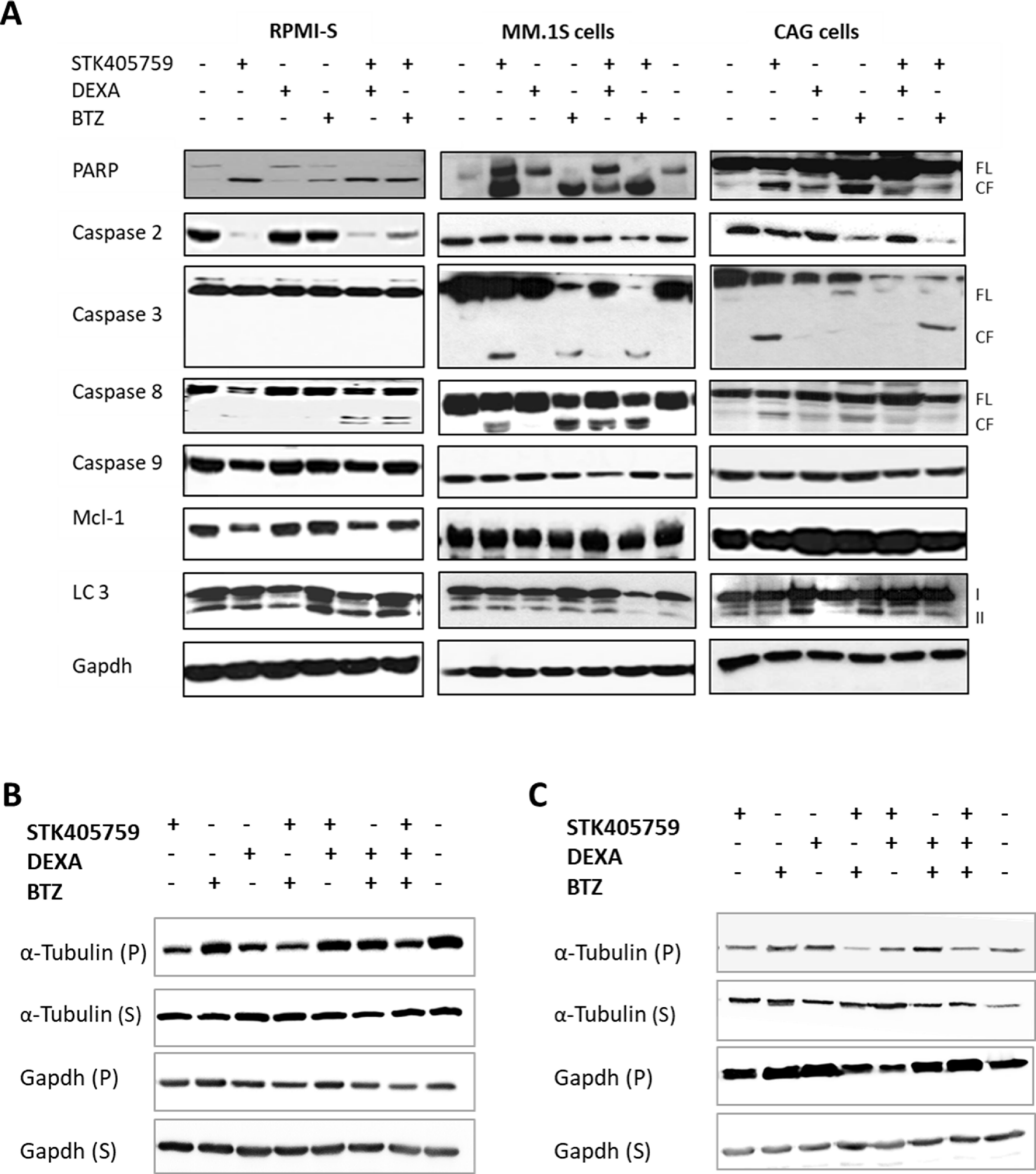
Combination of STK405759 with DEXA or BTZ induced caspase activation in a cell specific manner and increased tubulin depolymerization (**A**) RPMI-S, MM.1S and CAG cells were treated with STK405759, DEXA and BTZ alone or in combination with STK405759 for 24 h. Protein lysates from treated cells were immunoblotted using anticaspase-2, -3, -8, -9, PARP, mcl-1, LC3 and gapdh antibodies. FL, and CF indicate the full length and cleaved forms, respectively. (**B**–**C**) Tubulin and gapdh in the polymerized (P) and soluble (S) fractions of MM cells were analyzed by immunoblotting in RPMI-S and CAG treated cells. Blots are representative of two-three independent experiments.

In order to elucidate the cellular mechanisms driving the advantage of combined therapy observed on viability, we tested the expression level of proteins related to cell death. Three MM cell lines (RPMI-S, MM.1S and CAG) were treated with STK405759 alone or in combination with DEXA or BTZ for 24 h and their proteins isolated and immunoblotted. The pattern of cell death pathway activation differed among the cell lines after STK405759, DEXA or BTZ treatment. Thus, STK405759 increased cleavage of caspase 3 in MM.1S and CAG, but not in RPMI-S cells, whereas caspase 2 activation and decreased myeloid cell leukemia-1 (mcl-1) expression levels occurred mainly in RPMI-S cells. Poly-(ADP-ribose) polymerase (PARP) and caspase 8 activation were common to all tested cells after STK405759 treatment. DEXA treatment activated PARP and microtubule-associated protein light chain 3 (LC3) in CAG cells, whereas BTZ activated caspase 8 on MM.1S cells, caspase 2 on CAG cells, and PARP on RPMIS, MM.1S and CAG cells. The combined treatments included activation of each of the proteins activated by the single agents alone, confirming the enhanced cytotoxicity of these combinations in cell viability assays (Figure 4A).

We showed that STK405759 inhibited tubulin polymerization in a concentration-dependent manner in an *in vitro* cell-free system and in MM cells [15].

Here we observed that neither DEXA nor BTZ affected the level of soluble or polymerized tubulin in RPMI-S cells. The combination of each of them with STK405759, resulted in reduced expression of polymerized tubulin (Figure 4B–4C).

### STK405759 combined with DEXA or BTZ exhibited significantly enhanced antimyeloma efficacy *in vivo*

Based on the efficacy of STK405759 combined with DEXA or BTZ in targeting MM cells *in vitro*, we validated these findings *in vivo*. Mice with a subcutaneous human MM tumor were treated with STK405759, DEXA, BTZ, STK405759+DEXA or STK405759+BTZ for 3 months.

As a single agent, DEXA was more potent than was STK405759 (*p* < 0.0001). This difference decreased from day 80 (*p* = 0.2) of treatment due to development of resistance in the DEXA treated group. Importantly, treatment with STK405759 and DEXA was significantly more effective than with DEXA alone (*p* = 0.002 on day 31 and *p* = 0.000001 on day 80, *t*-test), with reduced tumor burden in all the treated mice. The combination induced a clear inhibition of tumor growth, which was maintained at least for 90 days. On day 49, the tumors from DEXA treated mice relapsed and started to grow, reaching a tumor volume 5 times larger than that of the STK405759 + DEXA treated mice on day 80. Complete tumor regression was observed in 50% of the mice treated with STK405759 plus DEXA and remained stable for 60 days after the last treatment.

**Figure 5:**
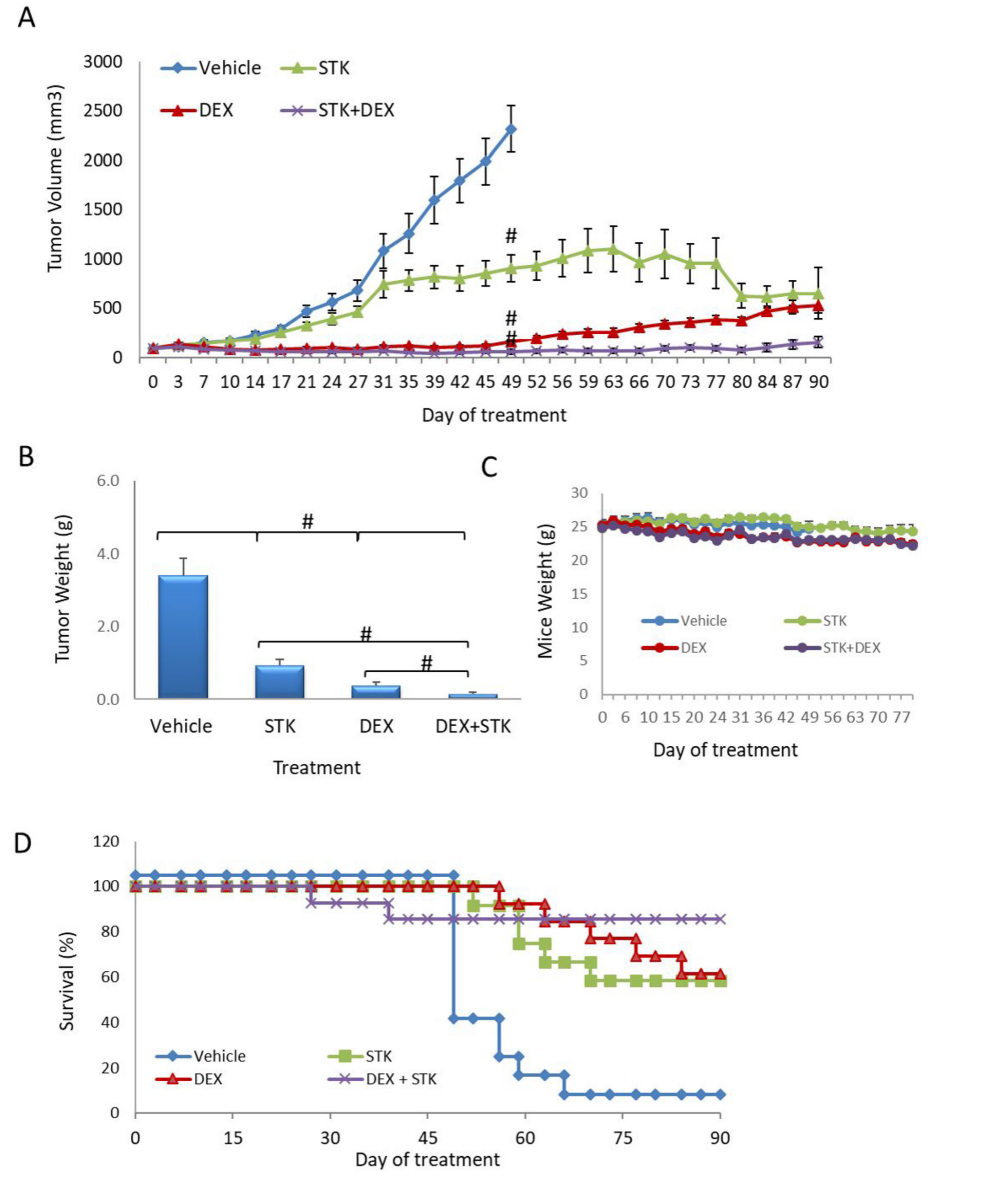
STK405759 in combination with DEXA decreased tumor growth in a MM xenograft mouse model SCID mice were inoculated subcutaneously RPMI-S cells and treated with STK405759 (0.5 mg/kg) or DEXA (1 mg/kg), or their combination. (**A**) Tumor burden was measured every 2–3 days using a caliper. (**B**) Tumor weight was measured when tumor size was 2500 mm^3^ or at the end of the experiment (**C**) Body weight was evaluated twice a week. (**D**) Kaplan–Meier curves of overall survival of mice treated with vehicle, STK405759, DEXA or STK405759/DEXA for 90 days. Data are presented as mean ± SE. ^#^*p* < 0.0001, *t*-test.

The improved efficacy of STK405759 combined with DEXA was confirmed by the weight of the extracted tumors at the end of the experiment (*p* = 0.007). DEXA plus STK405759 significantly improved survival relative to the vehicle-treated group (*p* < 0.0001) (Figure 5).

*In vivo*, the efficacy of BTZ and STK405759 as single drugs were similar (*p* > 0.5, *t*-test).

**Figure 6:**
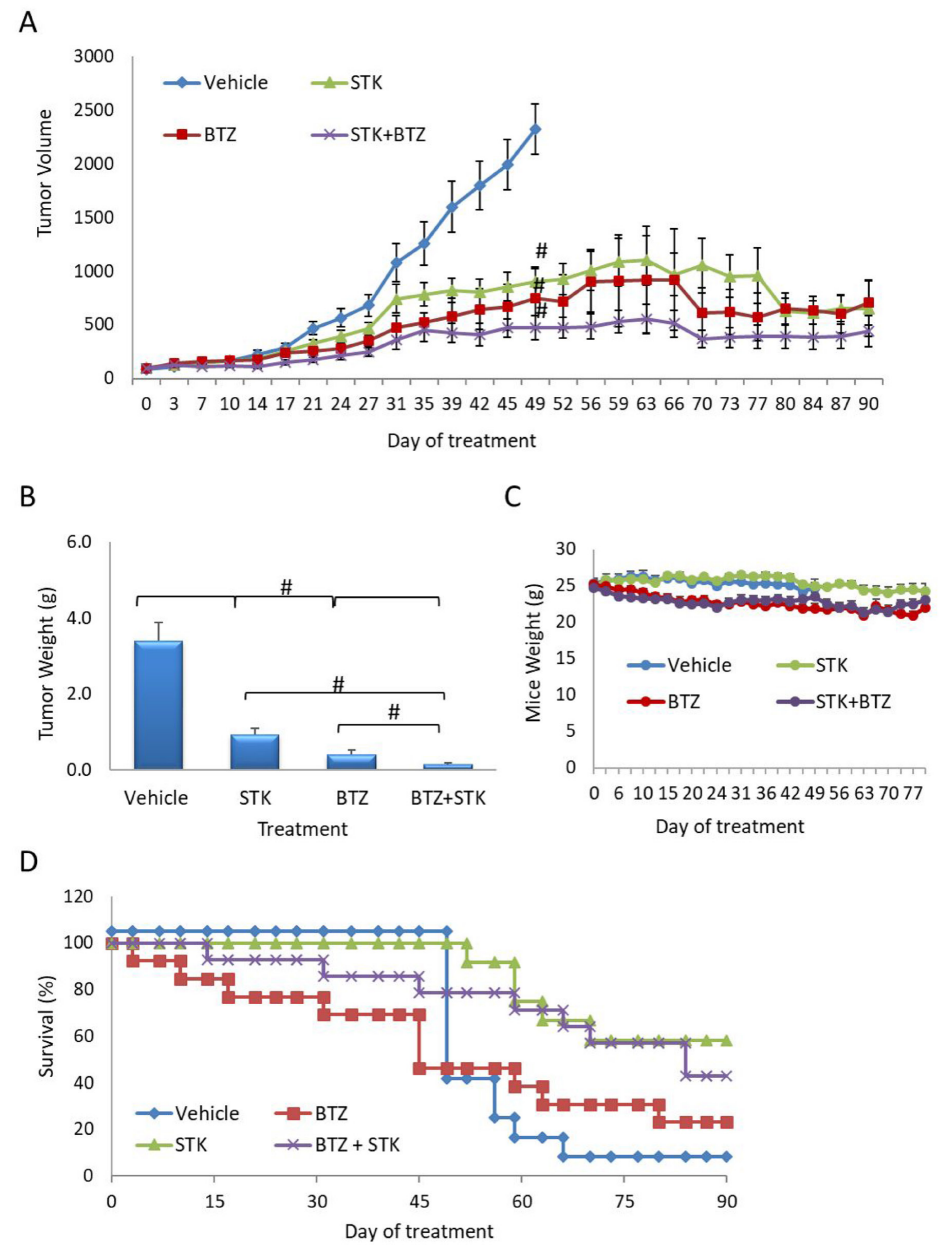
STK405759 in combination with BTZ decreased tumor growth in a MM xenograft mouse model SCID mice were inoculated subcutaneously with RPMI-S cells and treated with STK405759 (0.5 mg/kg) or BTZ (0.2 mg/kg), or their combination. (**A**) Tumor burden was measured every 2–3 days using a caliper. (**B)** Tumor weight was measured when tumor size was 2500 mm^3^ or at the end of the experiment. (**C**) Body weight was evaluated twice a week. (**D**) Kaplan-Meier curve of overall survival of mice treated with vehicle, STK405759, BTZ or STK405759/BTZ for 90 days. Data are presented as mean ± SE. ^#^*p* < 0.0001, *t*-test.

Combined BTZ and STK405759 treatment significantly reduced tumor size as compared to each component administered alone (*p* < 0.5, *t*-test). After 42 days of treatment with BTZ, STK405759 or BTZ + STK405759, tumor volume decreased ∼60%, ∼50% and ∼80%, respectively (Figure 6). The improved efficacy of STK405759 when combined with BTZ was also confirmed by the weight of the tumors extracted after the final tumor volume measurement (*p* < 0.05 *t*-test; Figure 6). With respect to toxicity, decrease in body weight was not observed in mice receiving combined treatments. STK405759 resulted in better survival than BTZ-treated mice (*p* = 0.036, chi-square). STK405759 combined with BTZ improved survival significantly, as compared to vehicle (*p* = 0.003, chi-square) and to a lesser extent in the groups treated with BTZ or STK405759 alone, respectively (Figure 6).

Importantly, blood tests revealed no toxicity-related complications with either combination tested. There were no signs of anemia, as evidenced by normal red blood cell, hemoglobin, hematocrit and platelet values relative to naïve mice, or of hepatic or renal failure (Supplementary Figures 1, 2), suggesting a favorable therapeutic index for these regimens.

### STK405759 has antiangiogenic activity and induces apoptosis *in vivo*

Tumor samples were analyzed with H&E staining to evaluate morphological changes and by TdT-mediated dUTP nick-end labeling (TUNEL) assays to evaluate apoptosis. Anti-CD34 monoclonal antibody was used to estimate microvessel density (MVD) in RPMI-S cells, *in vivo*. Tumors from control mice had typical histologic appearance, whereas those from mice treated with the drug combinations had lower MM cell density, as well as signs of necrosis.

**Figure 7:**
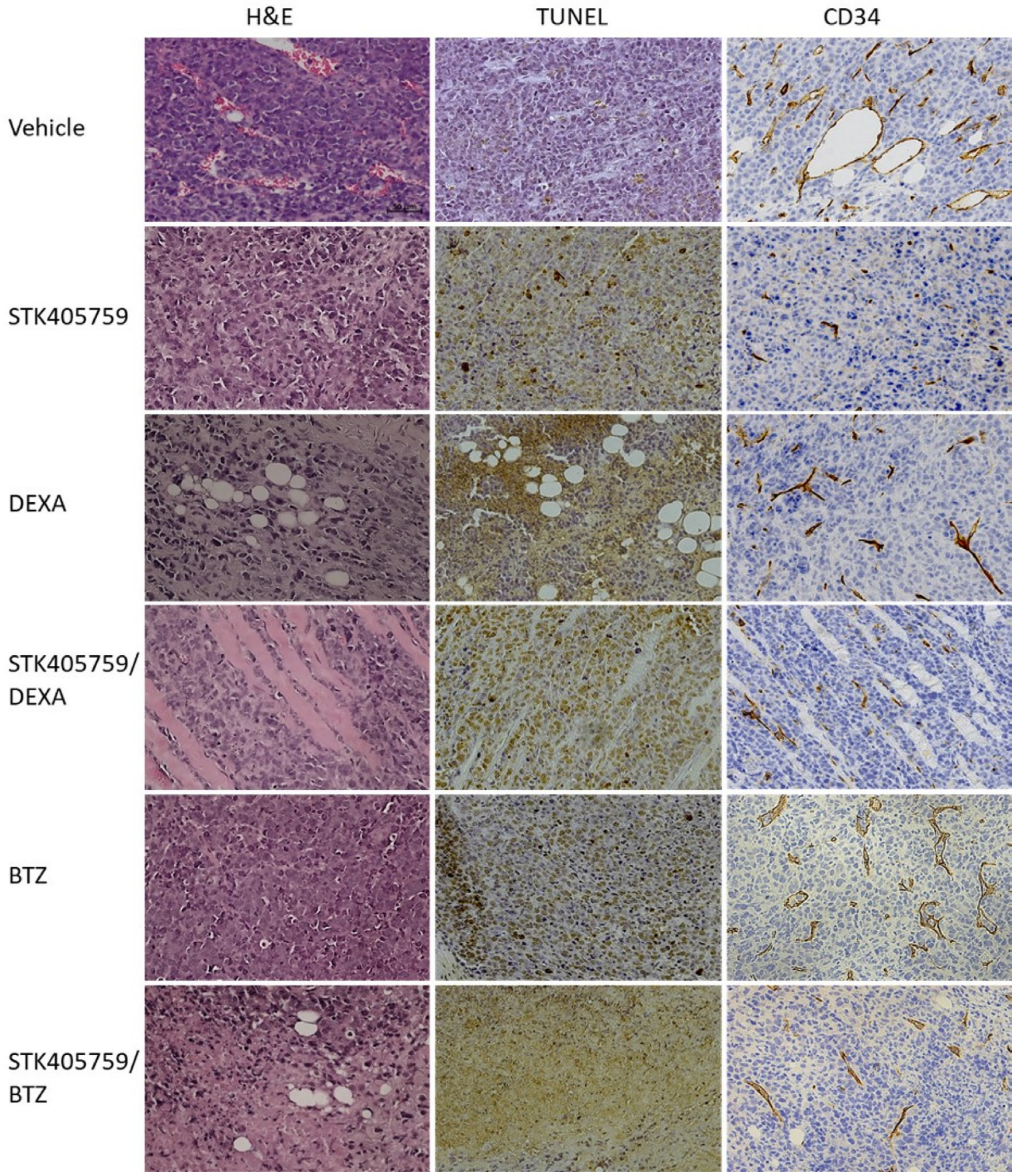
Effect of STK405759, DEXA, BTZ and their combinations on angiogenesis and tumor cell death Representative microscopic images of tumor sections from treated mice stained with HE, TUNEL or anti-CD34 antibody are shown. The slides were examined using Zeiss microscope, and images were processed using ZEN digital imaging software (Bar = 50 μm).

The combination of STK405759 with DEXA or BTZ increased the number of necrotic areas and of TUNEL-positive apoptotic tumor cells as compared to treatment with vehicle or either agent alone (Figure 7), confirming the activation of apoptosis observed in the *in vitro* results, discussed earlier.

STK405759 significantly decreased MVD after therapy in treated mice, supporting an antiangiogenic effect of STK405759 *in vivo*. The efficacy of STK405759 plus DEXA or BTZ was also enhanced by reduced vascular areas and fewer branched vessels in tumor tissues (Figure 7), with reduced expression level of CD34 endothelial stem cell markers.

## DISCUSSION

MM remains incurable in most patients, prompting an ongoing search for additional therapeutic options, including multidrug-combinations that induce long-term tumor regression with little cross-resistance and low toxicity.

Here, we showed that the combination of STK405759 and DEXA or BTZ increased cytotoxicity, as compared to each drug alone, through activation of each of the apoptotic-related proteins.

In an *in vivo* xenograft model, treatment with STK405759 combined with DEXA or BTZ resulted in significant inhibition of tumor growth, sustained tumor regression and increased overall response compared to treatment with each drug alone.

Bone marrow angiogenesis correlates with disease progression and prognosis in MM patients [17, 18]. The grade of vascularization before initiation of therapy was found to be a predictive factor for survival [19]. BTZ exhibits anti-angiogenic activity both *in vitro* and *in vivo* [20, 21] and MM patients treated with BTZ show a significant decrease in MVD [22]. Similarly, MTAs demonstrate antiangiogenic activity in a wide range of malignancies [23, 24]. In the current study, STK405759 disrupted existing blood vessels in xenograft tumors, acting both as a cytotoxic agent and as an anti-angiogenic drug. The anti-angiogenic effect of STK405759 might contribute to the increase efficacy observed after its combination with DEXA and BTZ *in vivo*.

Historically, vincristine has been used as part of conventional chemotherapeutic regimens for MM, as patients were treated with the combination of vincristine, doxorubicin and DEXA (VAD) [10, 25–28]. This combination therapy has been replaced in the last decade due to hematological (granulocytopenia and thrombocytopenia) and non-hematological toxicity (neurotoxicity and impaired cardiac function), modest activity, the development of chemoresistance and the emergence of novel therapeutic agents. The main mechanism of vincristine chemoresistance was through overexpression of the multidrug efflux pumps P-glycoprotein [14, 29, 30]. We found that the combination of STK405759 with DEXA or BTZ was not affected by this mechanism of resistance. In addition, blood tests of treated mice showed no sign of hematological toxicity.

Various groups reported that the use of vincristine combined with anthracyclines significantly reduced the antitumor effects, as compared with the administration of each drug alone [31–33]. In accordance with these results, we also found antagonistic activity between STK405759 and doxorubicin [15]. The antagonistic effect between MTAs and anthracyclines might explain the modest activity of historical combination therapy (e.g. VAD). Despite this antagonistic effect, a recent case report described a patient with secondary plasma cell leukemia refractory to BTZ-DEXA and to lenalidomide-DEXA therapies, who achieved partial remission after vincristine-doxorubicin-DEXA treatment, suggesting potential benefits of using MTA agents in the treatment of unresponsive patients [34].

In summary, we showed that the use of STK405759 with BTZ or DEXA significantly enhanced efficacy and increased survival in a MM mice model, as compared to each drug alone, and without observable side effects. These data provide a rationale for further clinical evaluation of STK405759in combination with DEXA or BTZ for patients with relapsing and refractory MM.

## MATERIALS AND METHODS

### Compounds

STK405759 was synthesized and provided by Mcule, Inc. Lab (Palo Alto, CA, USA). DEX was purchased from Sigma-Aldrich (St. Louis, MO, USA). BTZ was purchased from Apexbio Technology, Houston, TX, USA.

### Cell lines

Human MM cell lines RPMI 8226 (RPMI-S), MM.1S, MM.1R, U266 and the human bone marrow stromal cell (BMSC) line HS-5 were purchased from ATCC, Manassas, VA, USA. The RPMI sublines RPMI-MR20, RPMI-LR5 and RPMI-DOX40 and the CAG, OPM1 and OPM2 cell lines were kindly provided by Jana Jakubikova (Dana-Farber Cancer Institute, Boston, MA, USA). MM cell lines were grown in RPMI-1640 medium and HS-5 in Dulbecco’s modified Eagle medium (Gibco/BRL, Gaithersburg, MD, USA), both supplemented with 10% fetal calf serum and antibiotics (Biological Industries, Beit Haemek, Israel).

### Cell viability assay

MM cell lines were plated at 1–2 × 10^4^ cells per 96-well and treated with STK405759 (25 nM), DEXA (10 nM (MM.1S cells) and 100 nM, remaining cells), BTZ (3 nM), or their combination for 48 h. Cell viability was measured using XTT cell proliferation Kit (Biological Industries, Beit Haemek, Israel) according to manufacturer’s instructions.

### Co-culture experiments

RPMI-S cells, previously stained with 5-(and 6)-carboxyfluorescein diacetate succinimidyl ester (CFSE), (Thermo Fisher Scientific, Inc., Waltham, MA, USA), were added to wells seeded 24 h earlier with HS-5 BMSCs and exposed to STK405759 (30 nM), DEXA (10 nM (MM.1S cells) and 100 nM (RPMI-S and CAG cells), BTZ (7.5 nM) or their combination, for 48 h. Then, the cells were collected and stained with PI. Data were collected using FACS Calibur (Becton Dickinson Biosciences, San Jose, CA, USA) and analyzed with FlowJo software (FlowJo, LLC, USA).

### Immunoblotting analysis

For immunoblotting analyses, MM cell lines were plated in RPMI 1640 medium with 10% FBS and antibiotics. RPMI-S, MM.1S and CAG cells were treated with STK405759 (30 nM), DEXA (10 nM (MM.1S cells) and 100 nM (RPMI-S and CAG cells), BTZ (7.5 nM), and in combination with STK405759 for 24 h. Cells were lysed in RIPA lysis buffer containing 10 mM sodium pyrophosphate, 2 mM sodium orthovanadate, 5 mM sodium fluoride, 5 g/ml aprotinin, 5 g/ml leupeptin, and 1 mM phenylmethylsulfonyl fluoride (Sigma-Aldrich, St. Louis, MO, USA). Proteins were separated by sodium dodecyl sulfate-polyacrylamide gel electrophoresis, transferred onto nitrocellulose membranes and immunoblotted with anti-caspase-2, caspase-3, caspase-8, caspase-9, PARP, LC3 (Cell Signaling Technology, Beverly, MA, USA), mcl-1, and gapdh antibodies (Santa Cruz Biotechnology, CA, USA). Immunoreactive bands were detected by Western Blot chemiluminescence reagents (Thermo Fisher Scientific Inc, Waltham, MA USA) and exposed on X-Ray film (Fujifilm Corporation, Tokyo, Japan).

### Analysis of microtubule polymerization in MM treated cells

RPMI-S and CAG cells were treated and after 18 hours lysed in microtubule stabilizing buffer (20 mM Tris–HCl, pH 6.8, 0.14M NaCl, 1 mM EGTA, 0.5% NP-40, 1 mM MgCl2, 0.4 µg/ml paclitaxel, protease inhibitor mixture (Complete; Roche Diagnostics), protease inhibitor cocktail 1 and 3 (Sigma-Aldrich, St. Louis, MO, USA) and 1 mM phenylmethylsulfonyl fluoride) and centrifuged at 12,000 rpm for 10 min. The supernatants containing soluble tubulin and the pellets containing polymerized tubulin were collected and subjected to immunoblot analysis with anti-tubulin and anti-gapdh antibodies (Sigma-Aldrich, St. Louis, MO, USA).

### *In vivo* efficacy evaluation

SCID mice (6–8-weeks-old) were maintained in accordance with Institutional Animal Care Use Committee guidelines. Mice were gamma-irradiated (150 rads) using Cs137 γ-irradiator source and injected subcutaneously with MM cells suspended in PBS (7 × 10^6^/mouse), 24 h post-irradiation. Three weeks later, when palpable tumors developed (average tumor size at start of treatment was 90 mm^3^), mice were randomized into 7 groups (*n* = 12–14 mice/group), and the following treatment protocols implemented: Group 1: vehicle control (10% DMSO+ 5% Tween 80 in PBS), Group 2: STK405759 (0.5 mg/kg, intraperitoneal (ip), 5 days/week), Group 3: DEXA (1 mg/kg, ip, 5 consecutive days, weekly), Group 4: BTZ (0.2 mg/kg,ip, 2 days weekly), Group 5: STK405759 (0.5 mg/kg, ip, 5 days weekly) plus DEXA (1 mg/kg, ip, 5 consecutive days weekly); Group 6: STK405759 (0.5 mg/kg, ip, 5 days weekly) plus BTZ (0.2 mg/kg, ip, 2 days weekly) and Group 7: Naive mice (without tumor, *n* = 5) for blood tests.

Evaluation of efficacy included inhibition of tumor growth, survival, blood tests, animals’ vital signs and gross pathology. Tumor size was measured by caliper measurements of the longest perpendicular tumor diameters, every 3–4 days to estimate tumor volume, using the formula representing the 3-dimensional volume of an ellipse: 4/3× (width/2) exp(2× (length/2)). Blood was drawn on days 20 and 44 of treatment. The blood tests were performed by Tel Hashomer Core Equipment Facilities. Animals were sacrificed when the tumor reached 2.5 cm, or ≥20% decrease in body weight or appeared moribund, to prevent unnecessary morbidity to the mice. Overall survival, was defined as time between initiation of treatment and sacrifice or death. The Kaplan-Meier end-product analysis was used to compare control vs. each group of treated mice.

### Histochemistry

The tumors were fixed in 4% paraformaldehyde for 24 h (Sigma-Aldrich, St. Louis, MO, USA), washed with PBS, dehydrated in increasing alcohol concentrations and embedded in paraffin blocks. Sections were deparaffinized and rehydrated, treated with proteinase K (20 μg/ml) for 15 min and washed in PBS. Endogenous peroxidase was blocked with 3% hydrogen peroxide for 15 min. Fragmented nuclear DNA, associated with apoptosis in histological sections, was labeled *in situ* with digoxigenin-deoxyuridine (dUTP), introduced by terminal deoxynucleotidyl transferase (TdT), using ApopTag^®^ peroxidase *in situ* apoptosis detection kit according to manufacturer’s instructions (Intergen, Oxford, England, UK). The reaction was terminated using the ApopTag^®^ stop buffer followed by anti-digoxigenin-peroxidase application and the labeled nuclei were detected with ACE substrate as the chromogen (Sigma-Aldrich, St. Louis, MO, USA). MVD were labeled using anti-CD34 antibody (Abcam, ab81289, USA). The slides were examined using a Zeiss microscope, and images were processed using ZEN digital imaging software.

### Statistical analysis

The differences in drug-treated vs. control cultures was determined using Student’s *t*-test. Data are presented as mean ± standard error (SE). For *in vivo* experiments, survival was assessed using Kaplan-Meier curves and Chi-square analyses.

## SUPPLEMENTARY MATERIALS FIGURES



## Abbreviations

MM: multiple myeloma
BTZ: bortezomib
DEX: dexamethasone
mcl-1: myeloid cell leukemia-1
PARP: Poly-(ADP-ribose) polymerase
LC 3: microtubule-associated protein light chain 3
ip: intraperitoneal
TUNEL: TdT-mediated dUTP nick-end labeling
MVD: microvessel density
