# Supplementary material for: STK405759 as a combination therapy with bortezomib or dexamethasone, in in vitro and in vivo multiple myeloma models
Source: Oncotarget. 2018 Jul 31;9(59):31367–79. doi: 10.18632/oncotarget.25825 (PMC6101139; doi:10.18632/oncotarget.25825)
Supplement: Supplementary file 1 [file oncotarget-09-31367-s001.pdf]

STK405759 as a combination therapy with bortezomib or dexamethasone, in *in vitro* and *in vivo* multiple myeloma models

SUPPLEMENTARY MATERIALS

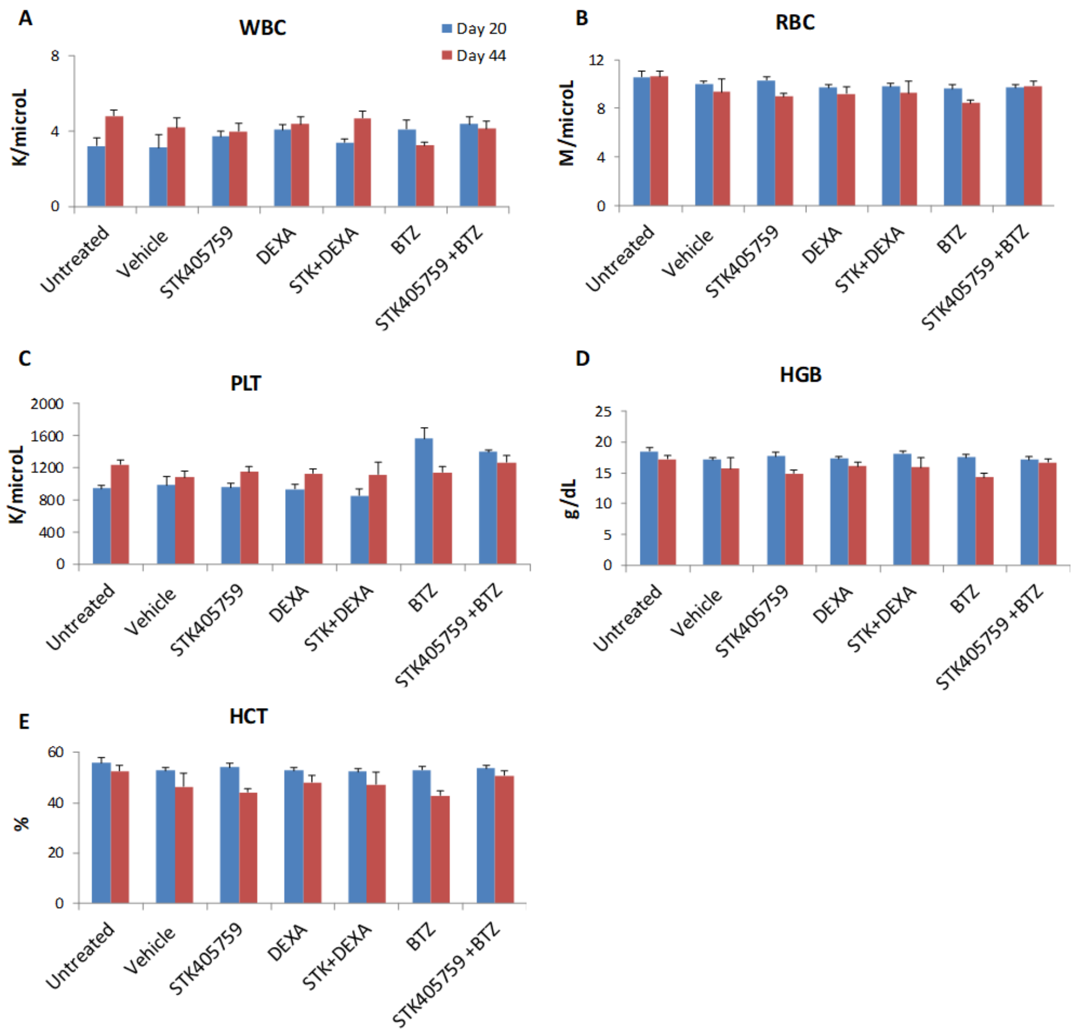

Supplementary Figure 1: Blood tests of mice treated with STK405759, DEXA, BTZ and their combinations in a MM xenograft model. Blood was drawn on days 20 and 44 from 7 mice in each treatment group and the following tests were performed: (**A**) white blood cell (WBC) count; (**B**) red blood cell (RBC) count; (**C**) platelet (PLT) count; (**D**) hemoglobin quantification, and (**E**) hematocrit determination.

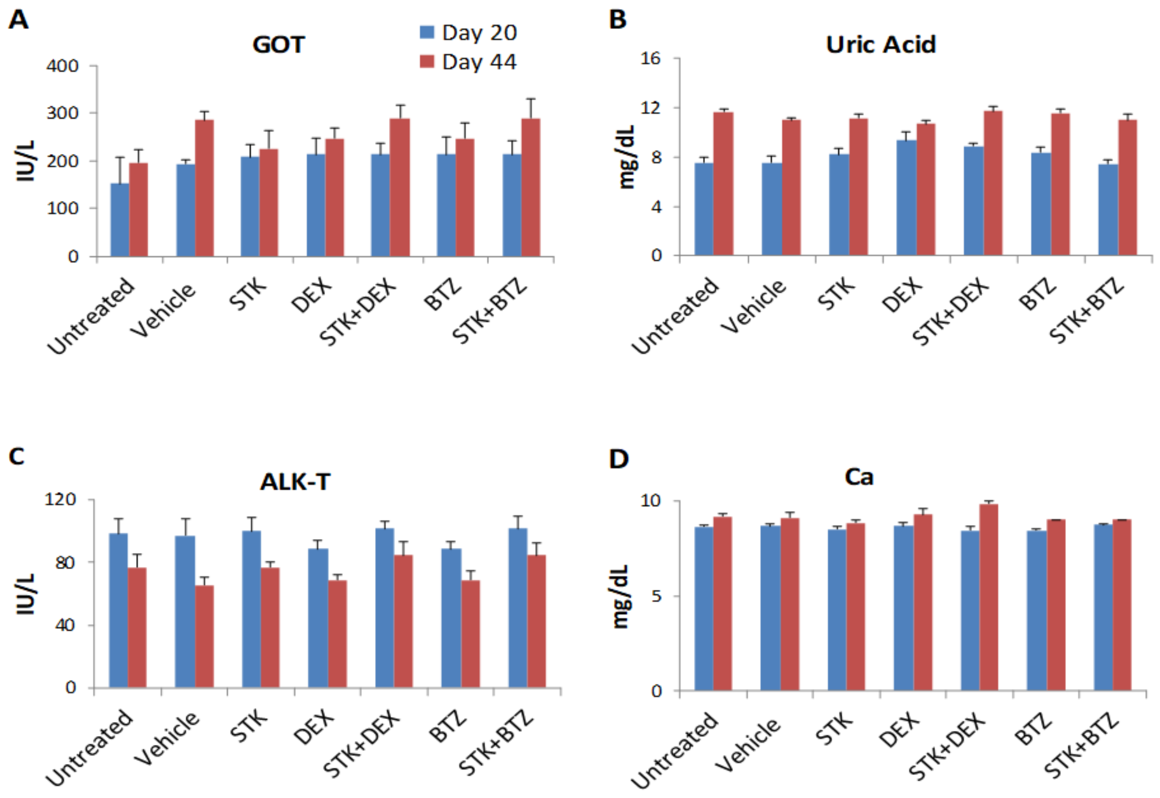

Supplementary Figure 2: Blood tests of mice treated with STK405759, DEXA, BTZ and their combinations in a MM xenograft model. Blood was drawn on days 20 and 44 from 7 mice in each treatment group and the following tests were performed: (**A**) aspartate aminotransferase (GOT), (**B**) uric acid, (**C**) alkaline phosphatase, and (**D**) calcium (Ca) determination.
